# Polytraumatization in an adult national sample and its association with psychological distress and self-esteem

**DOI:** 10.1002/brb3.298

**Published:** 2014-12-04

**Authors:** Doris Nilsson, Örjan Dahlstöm, Gisela Priebe, Carl Göran Svedin

**Affiliations:** 1 Section for Psychology, Department for Behavioral Sciences and Learning, Linköping UniversityLinköping, SE-581 83, Sweden; 2 Section for Disability Research, Department for Behavioral Sciences and Learning, Linköping UniversityLinköping, SE-581 83, Sweden; 3 Department of Psychology, Linnæus UniversityVäxjö, SE-35195, Sweden; 4 Child and Adolescent Psychiatry, Department of Clinical and Experimental Medicine, Faculty of Health Sciences, Linköping UniversityLinköping, SE-581 85, Sweden

**Keywords:** Adults, anxiety, depression, polytraumatization, self-esteem

## Abstract

**Objective:**

The objective of this study was to examine the prevalence of self-reported experiences of potential childhood traumas and polytraumatization, and to find cut-off values for different kinds of potential traumatic events in a national representative sample of adults in Sweden. In addition, to analyse the association between polytraumatization and both psychological distress and global self-esteem.

**Method:**

A web-based survey - containing SCL-25 and Rosenberg Self-Esteem Scale, and Linköping Difficult Life Events Scale - Adult - was sent out to a nationally reprative sample and 5062 people chose to participate in the study.

**Results:**

Results showed that almost everyone (97%) has experienced at least one potential traumatic event and that polytraumatization (the 10% of the participants with most reported traumas) was significantly (*Z* = 12.57, *P* < 0.001, *r* = 0.18) associated with psychological distress and global self-esteem. Gender differences were significant (*Z* = 8.44, *P* < 0.001, *r* = 0.12), in that men experience more noninterpersonal traumas but women report more symptoms. The effect sizes regarding the impact of potential trauma on self-esteem were largest for women with experience of polytraumatization in the age group 18–25 (*r* = 0.48). There was almost linear increase in psychological distress and linear decrease in self-esteem with increasing number of traumatic events experienced.

**Conclusion:**

Experience of polytrauma can be considered an important factor to take into account in psychiatric settings as well.

## Significant Outcomes

- This study shows that it is common, in a normative adult population, to have experienced at least one potential childhood trauma.
- Cut-off values for polytraumatization were established concerning noninterpersonal, interpersonal, and adverse childhood circumstances. The impact of experienced potential self-reported trauma has larger effects on women.
- There is a linear association between polytraumatization and the increase of psychological distress and decrease of global self-esteem.

## Limitations

- The study has used retrospective data, and there is always a danger of recall bias.
- In this study, no specific trauma instrument has been used which might have given more trauma specific results.

## Introduction

There are an increasing number of studies that show an association between having experienced polytrauma - which means multiple types of potential traumas - and a broad variety of psychosocial and somatic health problems later on in life, both among children and adults (Maschi et al. 2012). In the research literature repeated or/and multiple types of potential trauma, have been labeled differently i.e. cumulative trauma, polyvictimization, and polytrauamtization (Scott-Storey 2011).

Most of the studies have been conducted in adolescent populations (Finkelhor et al. 2007b; Nilsson et al. 2010, 2012; Soler et al. 2012, 2013; Zetterqvist et al. 2012) and Finkelhor and coworkers have, in several studies, demonstrated the negative impact of polyvictimization (*multiple types of victimizations*) among adolescents (Finkelhor et al. 2007a,b, 2009a; Turner et al. 2010). Finkelhor and colleagues have identified victims and polyvictims by counting the different types of youth victimizations over both the last year and the (youth) lifetime, and have suggested classifying polyvictims as the 10% most victimized in the population (Finkelhor et al. 2009a).

Among adult studies of childhood trauma, the large Adverse Childhood Experience studies (ACE) have been at the forefront of this research for many years (Feletti et al. 1998). The ACE-studies have shown us how adverse childhood experiences such as: Abuse*: emotional, physical, and sexual,* Neglect: *emotional and physical* and Dysfunctional Family/Household: *mother was physical abused, drugs in family, mental illness, divorce, somebody in the family in prison,* are clearly associated with psychosocial as well as worsened somatic health in adult life (Feletti et al. 1998; Anda et al. 1999, 2001, 2002; Dietz et al. 1999; Dube et al. 2001a,b, 2003; Dong et al. 2003, 2004).

Other researchers have also recognized the cumulative effect of experiences from different types of potentially traumatic life events, some studies from community samples (Chiara and Straus 2008; Widom et al. 2008; Richmond et al. 2009) and some from clinical populations (Briere et al. 2008; Cloitre et al. 2009). In the studies by Chiara and Straus (2008) and Richmond et al. (2009) polyvictimization accounted for a significant proportion of the variability on scores of psychological distress and also for unique variance.

It has also been shown that there is an increased risk of revictimization after a person has been victimized once (Noll et al. 2003; Finkelhor et al. 2007b; Widom et al. 2008).

To experience one potential trauma is not uncommon in adolescent populations (Finkelhor 2008; Finkelhor et al. 2009a) as well as in adult populations (Arata et al. 2005; Richmond et al. 2009). Richmond and colleagues (Richmond et al. 2009) found in two study samples that exposure to at least one individual type of potential trauma was reported by 98%, and almost half of the population 40–49% reported at least one in five of the categories (property crime, physical assault, child maltreatment, peer/sibling victimization, sexual victimization, and witnessed indirect victimization).

Both to be a polyvictim and to have experienced polytrauma have been shown to have greater predictive value on mental health than single or one type of traumas even if repeated (Chiara and Straus 2008; Briere et al. 2008; Cloitre et al. 2009; Chartier et al. 2010), also on the effects on symptom complexity (Briere et al. 2008; Cloitre et al. 2009) and self-esteem (Soler et al. 2012, 2013). However, even if the experience of polytrauma in the above mentioned studies was shown to have the greatest predictive value on mental health it was also shown that physical abuse and sexual abuse, including rape, also predicted poor health, but to a lesser extent than polytrauma (Chiara and Straus 2008; Briere et al. 2008; Cloitre et al. 2009; Chartier et al. 2010).

Even if there is strong evidence for the negative effects of experiences of trauma and polytraumatization we need to know more about this in normative samples and different cultures. It is important to look at the prevalence of childhood trauma and its consequences in the ages between 18 and 65. It is also essential for research to cover the broad spectrum of potential traumas such as: noninterpersonal (nIPE), interpersonal (IPE), and adverse childhood circumstances (ACC) and the effects of polytraumatization (PT), otherwise the results will be too narrowly interpreted. Since there is a lack of studies concerning polytraumatization in adult populations and in order to highlight the above mentioned aspects of potential traumatic events in a representative adult population, this study was carried out.

## Aims of the Study

This study aims to explore the prevalence of self-reported potential traumatic events before the age of 18, in different age groups in a representative Swedish adult population and to identify cut-off values for self-reported experienced trauma of both noninterpersonal and interpersonal character, and adverse childhood circumstances.

A second aim was to investigate the interactions and associations between polytraumatization, psychological distress (*anxiety and depression*), and global self-esteem and also to look at possible education and gender differences.

## Materials and Method

This paper used data derived from a large representative sample of the Swedish population.

The epidemiological study was carried out in 2011 within the project: “Prostitution in Sweden. *Mapping and evaluation of the three Swedish prostitution units for support to people who are selling or buying sex and experiences and attitudes in the general population*”. The project consisted of eight parts, of which this study was one, and took place between the years 2009 and 2012 and resulted in a main report to the Swedish government (Svedin et al. 2012).

### Participants

From a national web panel of 71,446 people between 18 and 65 years old, a stratified representative sample from the Swedish population was drawn. This sample consisted of 9999 people who were invited to participate. Of these 4215 did not answer, 701 started to answer but did not complete the questionnaire, 12 refused and 5071 chose to participate. The participating rate of 50.7% is in line with what has been obtained in former national studies (Lewin 1998).

## Procedure

The survey consisted of 81 questions. The participants were asked about their experience of buying or selling sex as well as potential traumatic childhood experiences and different aspects of psychological well-being. The survey included several standardized scales. In this study the following questionnaires were used.

## Questionnaires

### Linköping's youth life events scale- adult, LYLES -A

The Linköping's Youth Live Events Scale –Adult (LYLES-A) is a recently developed trauma history inventory (Nilsson et al. 2010), intended to cover several important areas of potentially traumatic events and circumstances during childhood, up to the age of 18. It contains 23 main questions and 18 more detailed secondary items, making a total of 41 questions. Eighteen items are designed to identify noninterpersonal (nIPE) traumas, 13 items identify interpersonal (IPE) traumas and 10 items ask questions about more enduring Adverse Childhood Circumstances, (ACC), see Table 1 for the whole scale. There are subquestions on several items to identify the respondent's proximity to the event, i.e. whether the person has experienced the event him/herself, seen it or just heard about it. The test–retest reliability has been found to be *r* = 0.79 (*P* < 0.01) and kappa statistics (*Cohen's kappa*) item per item range between 0.44 and1.0 (Finkelhor et al. 2007b). This is the first time the LYLES has been used in a population based group with adults. The response rate of the LYLES in this study was 99.8%, i.e. only nine participants answered “Don't want to answer” to (all of) the LYLES questions.

**Table 1:** Reported potentially traumatic life events on LYLES in percent, for men and women and different age-groups

Question	Men					Women					Men and Women
	18–25 (*n* = 177)	26–39 (*n* = 672)	40–49 (*n* = 557)	50–65 (*n* = 1139)	18–65 (*n* = 2545)	18–25 (*n* = 336)	26–39 (*n* = 747)	40–49 (*n* = 568)	50–65 (*n* = 997)	18–65 (*n* = 2648)	18–65 (*n* = 5193)
nIPE											
Have you been in a car accident?	15.3	18.0	28.2	30.3	25.5	17.3	15.5	20.4	22.0	19.2	22.3
Have you witnessed a car accident where you were not involved?	27.1	31.5	40.9	50.7	41.8	21.7	22.5	25.5	31.0	26.1	33.8
Has anyone in your family been in a car accident (without you being there)	24.9	24.4	25.3	24.6	24.7	25.3	22.2	19.2	22.0	21.	23.2
Has anybody else close to you been in a car accident (without you being there)?	22.0	20.7	24.6	24.2	23.2	23.8	16.2	16.0	16.9	17.4	20.2
Have you been in another accident?	29.9	28.1	30.2	26.3	27.9	25.3	15.7	12.9	15.7	16.3	22.0
Have you witnessed another accident where you were not involved?	29.4	28.0	31.2	33.9	31.4	25.6	15.8	16.9	18.2	18.2	24.7
Has anyone in your family been in another accident (without you being there)?	29.9	25.6	23.0	20.4	23.0	31.0	22.4	16.0	19.4	21.0	22.0
Has anybody else close to you been in another accident (without you being there)?	26.6	22.0	23.3	22.5	22.8	27.7	18.1	13.9	15.0	17.3	20.0
Have you been in hospital?	36.2	42.0	42.5	55.8	47.9	35.4	37.8	43.0	49.6	43.1	45.4
Has anyone in your family been in hospital?	50.8	60.9	60.1	66.3	62.4	68.5	59.2	57.9	66.2	62.7	62.6
Has anybody close to you been in hospital?	44.1	47.6	47.6	51.5	49.1	56.3	43.1	41.2	46.9	45.8	47.4
Has anybody in your family died?	30.5	37.1	46.1	48.8	43.9	33.9	36.7	46.1	51.7	44.0	43.9
Has anybody close to you died?	42.9	46.1	53.5	51.4	49.9	56.5	50.7	47.4	53.1	51.6	50.8
Have you been in a fire?	4.0	6.4	9.5	11.7	9.3	4.5	4.8	5.6	7.3	5.9	7.5
Have you witnessed a fire in another house?	18.6	26.2	33.4	46.0	36.1	15.5	18.3	20.8	26.7	21.6	28.7
Have you experienced a natural disaster?	5.1	2.5	4.1	5.0	4.2	2.7	1.7	3.0	4.3	3.1	3.6
IPE											
Have you been beaten or wounded by an adult in your family?	4.0	10.3	15.1	14.9	13.0	8.6	11.8	15.5	17.8	14.4	13.7
Have you been beaten or wounded by another person?	15.3	20.8	18.9	19.0	19.2	9.2	10.4	7.4	8.5	8.9	13.9
Have you witnessed anyone in your family (mother, sibling) been beaten or wounded by an adult in your family?	4.5	5.8	14.0	11.2	9.9	9.5	11.2	12.0	12.8	11.8	10.9
Have you witnessed anybody else been beaten or wounded?	21.5	28.4	31.4	31.0	29.7	18.2	13.8	13.9	18.2	16.0	22.7
Have you been bound or locked up against your will?	3.4	3.7	4.5	6.2	5.0	2.1	4.0	3.5	4.3	3.8	4.4
Have you been exposed to sexual acts against your will by an adult in your family?	0.0	0.4	1.1	0.8	0.7	0.9	1.1	2.5	2.5	1.9	1.3
Have you been exposed to sexual acts against your will by another person?	0.6	3.4	3.2	4.5	3.7	8.3	8.8	13.1	9.5	9.9	6.8
Have you witnessed anybody else getting exposed to sexual acts against their will?	0.0	1.0	1.4	1.3	1.2	0.6	0.9	0.7	1.4	1.0	1.1
Have you been threatened that anybody will harm you or somebody you care for?	20.9	21.8	16.5	14.7	17.4	14.6	12.6	11.1	8.8	11.1	14.2
Have you been robbed?	7.9	7.5	5.6	5.5	6.2	2.7	2.8	2.1	3.6	3.0	4.5
Have you been present when anybody else has been robbed?	6.8	3.7	5.0	4.2	4.4	6.0	2.5	1.6	3.0	3.0	3.7
Have you been home when anybody committed burglary?	2.8	2.1	2.2	3.1	2.6	3.9	1.7	2.1	2.1	2.2	2.4
Have you come home after a burglary?	5.1	6.7	9.0	10.2	8.7	7.1	7.8	7.2	6.9	7.3	7.9
nIPE											
Have you been in a war where you have heard or seen bombings or firings?	1.1	1.6	1.6	2.5	2.0	0.3	0.1	0.5	0.5	0.4	1.2
Have you escaped from your native country?	1.7	0.9	0.5	1.0	0.9	0.3	0.3	0.5	0.5	0.4	0.7
ACC											
Have you been bullied	39.0	38.5	36.7	30.0	34.3	40.8	34.5	36.2	31.7	34.6	34.5
Have you against your will, been separated from your parents to live in another place?	2.8	2.4	4.3	6.4	4.6	2.1	1.9	3.9	7.8	4.6	4.6
Have you been emotionally abused (e.g disparaged, humiliated)?	17.0	18.6	18.9	14.1	16.6	29.5	21.4	19.4	19.3	21.2	18.9
Have your parents got a divorce during your upbringing?	20.9	24.1	23.4	14.3	19.3	27.4	27.2	22.9	15.5	21.9	20.7
Have your parents quarreled a lot after the divorce?	35.1	29.6	31.5	27.6	29.9	39.1	34.0	30.0	31.2	33.2	31.7
Have your parents had problems with alcohol or other drugs during your upbringing?	7.3	13.4	15.8	14.5	14.0	13.1	16.1	16.1	15.3	15.4	14.7
Have your parents had mental problems health problems during your upbringing?	6.8	9.7	11.5	7.6	8.9	17.0	13.8	15.4	12.5	14.0	11.5
Do you, or have you had, a prolonged illness or handicap during your upbringing?	9.0	3.7	4.3	5.0	4.8	8.0	6.4	4.4	4.5	5.5	5.2
Do your parents have, or have they had, a prolonged illness or handicap?	11.3	9.5	9.7	13.1	11.3	15.2	13.0	12.6	11.9	12.9	12.1
Has anyone of your parents been in jail during your upbringing?	2.8	1.0	2.3	2.0	1.9	1.5	1.2	0.9	1.1	1.1	1.5

nIPE, noninterpersonal; IPE, interpersonal; ACC, adverse childhood circumstances.

In this study, the number of potentially traumatic experiences was summarized up in an index Polytraumatization (PT). For each of the aspects (nIPE, IPE, and ACC) on the LYLES-A, total score, nIPE, IPE, and ACC the 90th percentile was set as a limit for polytraumatization (PT) and initial analyses were based on three groups: (a) no trauma at all, (b) at least one trauma but no PT, and (c) PT. Further analyses were based on two groups: (a) no PT (nPT) and (b) PT.

### The Hopkins symptom check list-25

The Hopkins Symptom Check List-25 (SCL-25) is a self-administrated instrument widely used for the assessment of psychological distress. SCL-25 is developed out of SCL-90 and is one of the shortened versions (Derogatis et al. 1976). SCL-25 consists basically of two (anxiety and depression) of the nine original symptom dimensions of SCL-90 (Derogatis et al. 1974). The scale has been used in several cultural settings as well as psychometrically investigated and has proved to have psychometrically satisfactory characteristics, such as validity and reliability (Nettlebladt et al. 1993; Moreau et al. 2009; Strand et al. 2003). SCL-25 has 25 items on a four point Likert-scale ranging from 1 = “not at all” to 4 = “extremely”. Based on several studies an average item score of 1.75, calculated by dividing the total score by the number of items answered has been recommended as a valid predictor of clinical psychological distress - especially concerning depression but also anxiety (Strand et al. 2003). Cronbach's alpha in this study was *α *= 0.95.

### Rosenberg self-esteem scale

The Rosenberg Self- Esteem Scale (RSES) is a widely used scale for the measurement of global self-esteem. It was developed by Rosenberg (1965) with self-esteem as a one-dimensional concept that reflects a positive or a negative orientation toward the self. The psychometric qualities have been investigated in several studies and cultures (Rosenberg 1965). Psychometric studies have supported a one-dimensional scale approach (Hatcher and Hall 2009) but there are also studies proposing that the RSES is two-dimensional (Schmitt and Alllik 2005; Hatcher and Hall 2009; Marsh et al. 2010; Mullen et al. 2013). In this study, we have chosen the one-dimensional approach with reference to the study by Schmitt and Alllik (2005). The RSES has ten items, five positively and five negatively worded items. There are four possible answer choices from 3 = strongly agree to 0 = strongly disagree, a score is derived by reversing the five negative items and summing them with the five positive - one gets values between 0 and 30, high values are considered to be good self-esteem. Cronbach's alpha in this study was found to be *α* = 0.89. There is no cut-off point in the research literature presented for RSES (The Morris Rosenberg Fondation 2011).

## Statistical analyses

### LYLES-A; gender, age, and education

LYLES-A was considered in four different aspects (total, IPE, nIPE, and ACC). For each condition the 90th percentile was set as a limit for polytraumatization (PT) and participants were organized in three groups thereafter: (a) no trauma at all, (b) at least one trauma but no PT, and (c) PT. Thereafter, the group variable was put into a log-linear analysis together with gender (woman, man), age group (18–25, 26–39, 40–49, 50–65), and education (junior high school, high school, and university degree) in order to examine differences in distribution over different categories. Significant interactions were further analysed using Chi-square-statistics. For significant differences in distribution of PT, odds ratios and corresponding 95% CIs are reported. In further analyses two groups (nPT and PT) were used.

The distribution of Rosenberg total scores was negatively skewed and the distribution of SCL-25 scores was positively skewed and group comparisons between men and women (for nPT and PT, respectively) and between nPT and PT (for men and women, respectively) were made by Mann–Whitney *U* (comparing two groups) and Kruskal–Wallis (comparing more than two groups) tests. Due to the large sample size, even small differences were expected to be significant and therefore the effect size *r* is reported (*r *=* *0.1: small effect; *r *=* *0.3: moderate effect; *r *=* *0.5: large effect).

Differences in distribution of PT and nPT between distressed and nondistressed participants were examined using chi-square-statistics.

## Results

### Descriptives

For the total distribution of the different potential traumas on the LYLES-A over different age groups and gender, see Table 1. The most common events among both men and women were of nIPE character such as “Has anyone in your family been in hospital?” “Has anyone close to you died?” “Has anybody close to you been in hospital?” “Have you been in hospital?” and “Has anybody in your family died?” all endorsed by more than 40 percent of the sample. The most common endorsed IPE question was “Have you witnessed anybody else been beaten or wounded?” (men 29.7 and women 16.0 percent). Finally, “Have you been bullied?” was the most common circumstance among ACC endorsed by 34.5 percent, almost equal among the genders. 97% of the participants reported at least one potential trauma.

### Gender specific cut-off values

Log-linear analysis resulted, after elimination of nonsignificant higher-order effects, in a small but significant two-way interaction between PT and gender, *χ*^2^ (2, *N* = 5062) = 31.31, *P *<* *0.001, *Cramer's V *=* *0.08. In the PT group there were unexpectedly many men (std. residual = 3.9) and unexpectedly few women (std. residual = −3.8), but no such differences for the two groups who were not polytraumatized (nPT). Further analyses were therefore based upon nPT and PT groups. Due to differences in gender distribution among the polytraumatized, gender specific cut-off values for PT, i.e. the 90th percentiles (by definition the 10% who reported most traumas) were estimated, Table 2.

**Table 2:** 90th percentiles (as limit for *polytraumatization, PT*) for reported potential traumatic events on the LYLES for men (*n* = 2511) and women (*n* = 2472)

Age (years)	Men				Women			
	Tot	IPE	nIPE	ACC	Tot	IPE	nIPE	ACC
18–25	13	3	10	3	14	3	9	3
26–39	14	3	10	3	13	3	8	3.3
40–49	16	4	11	3.6	13	3	8	4
50–65	15	4	11	3	13	3	9	3

Tot, total number of trauma; n-IPE, noninterpersonal; IPE, interpersonal; ACC, adverse childhood circumstances.

### Differences in gender, age, and education

#### LYLES-A, total scale

The 90th percentile was set to 14 reported potential traumas. There were significant differences in distribution for women and men over different groups of trauma, *χ*^2^ (2, *N *=* *5062) = 31.31, *P *<* *0.001. The odds ratio of PT between men and women was 1.38, 95% CI [1.38, 1.97], i.e. for every 100 PT women there are 138 PT men.

There were also significant differences in distribution for different educational levels over different groups of trauma, *χ*^2^ (4, *N *=* *5024) = 14.18, *P *=* *0.007. Low educational levels were more often associated with PT than high educational level (odds ratio = 1.62, 95% CI [1.17, 2.25]), i.e. for every 100 PT persons with university degrees there were 162 PT persons with only junior high school.

#### LYLES-A, nIPE

The 90th percentile was set to 10 reported potential noninterpersonal traumas (nIPE). There were significant differences in distribution for women and men over different groups of potential traumas, *χ*^2^ (2, *N *=* *5061) = 93.53, *P *<* *0.001. The odds ratio of nIPE- PT between men and women was 2.51 95% CI [2.07, 3.04], i.e. for every 100 nIPE-PT women there are 251 nIPE-PT men. There were also significant differences in distribution for different educational levels over different groups of potential traumas, *χ*^2^ (4, *N *=* *5023) = 10.55, *P *=* *0.032, indicating higher levels of nIPE- polytraumatization with lower educational level. These differences were small (Cramer's *V* = 0.03).

#### LYLES-A, IPE

The 90th percentile was set to three reported potential interpersonal traumas (IPE). There were significant differences in distribution for women and men over different groups of trauma, *χ*^2^ (2, *N *=* *5056) = 31.71, *P *<* *0.001. The odds ratio of IPE-PT between men and women was 1.50, 95% CI [1.29, 2.75], i.e. for every 100 IPE-PT women there are 150 PT men. There was no interaction with educational level.

#### LYLE-A, ACC

The 90th percentile was set to three reported potential adverse childhood circumstance traumas. The ACC questions of LYLES-A did not interact with any of gender, age group, or educational level, i.e. there were no significant differences in distribution of traumatization and nontraumatization between different age-groups or gender.

### Psychological distress (SCL-25) by gender and age groups

SCL-25 scores were significantly higher for women (Median = 35) than for men (Median = 31), *Z *=* *12.37, *P *<* *0.001, *r *=* *0.17. SCL-25 scores were also significantly different between the age groups, *H*(3) = 127.33, *P *<* *0.001, the older the lower values of SCL-25 scores (18–25: *Mdn *= 34; 26–39: *Mdn *= 30; 40–49: *Mdn *= 29; 50–65: *Mdn *= 29; no significant differences between 40–49 and 50–65 though). If the recommended cut-off point ≤1.75 of SCL-25 for identification of clinically distressed individuals is used, 11.2% was found to be clinically distressed, in the total sample, for women it was 14.2% and men 8.1% (Table 3).

**Table 3:** Comparisons of SCL-25 scores between poly- (PT) and nonpolytraumatized (nPT) (for men and women separately) and between men and women (for poly (PT) - and nonpolytraumatized(nPT) separately)

Age	Men (PT vs. nPT)	Women (PT vs. nPT)	PT (men vs. women)	nPT (men vs. women)
Tot				
*18*–*65*	*0.16*	*0.20*	*0.20*	*0.20*
18–25	0.20	0.23	0.15^ns^	0.18
26–39	0.11	0.21	0.26	0.16
40–49	0.21	0.21	0.14	0.14
50–65	0.17	0.20	0.16	0.15
nIPE				
*18*–*65*	*0.08*	*0.10*	*0.18*	*0.17*
18–25	0.23	0.03^ns^	0.22^ns^	0.21
26–39	0.01^ns^	0.14	0.31	0.16
40–49	0.12	0.11	0.12^ns^	0.14
50–65	0.09	0.09	0.12	0.15
IPE				
*18*–*65*	*0.18*	*0.22*	*0.18*	*0.17*
18–25	0.14^ns^	0.27	0.27	0.16
26–39	0.15	0.24	0.24	0.16
40–49	0.24	0.25	0.15^ns^	0.13
50–65	0.18	0.20	0.11^ns^	0.14
ACC				
*18*–*65*	*0.21*	*0.25*	*0.26*	*0.17*
18–25	0.12^ns^	0.22	0.28	0.16
26–39	0.23	0.26	0.27	0.17
40–49	0.19	0.23	0.24	0.14
50–65	0.21	0.23	0.21	0.14

nIPE, noninterpersonal event; IPE, Interpersonal event; ACC, adverse childhood circumstances; *r*, effect size; ns, nonsignificant (*P* > 0.05). SCL-25 scores for all ages (18-25) are in italics. Comparisons are reported by their corresponding effect size *r* (small: *r* ∽0.1; moderate *r* ∽0.3; large: *r* ∽0.5).

### Distress and PT

There were significantly more reported potential traumas for participants who were above the cut-off point for being clinically distressed (average SCL-25 ≥1.75). Total trauma – Mdn = 9 versus Mdn = 6, *Z *=* *12.57, *P* < 0.001, *r* = 0.18. IPE trauma – Mdn = 1 versus Mdn = 0, *Z *=* *11.67, *P* < 0.001, *r* = 0.16. nIPE trauma – Mdn = 5 versus Mdn = 4, *Z *=* *6.6, *P* < 0.001, *r* = 0.09. ACC trauma – Mdn = 2 versus Mdn = 1, *Z *=* *14.56, *P* < 0.001, *r* = 0.20.

There was a significant difference in distribution of distressed participants between n-PT and PT, *χ*^2^ (1, *N* = 5018) = 138.91, *P *<* *0.001, *Cramer's V *=* *0.17, i.e. a low association between distress and PT. There was higher amount of distressed among PT (146 observed versus 61 expected, std. residual = 10.8). The pattern was consistent over gender and different age groups.

### Self-esteem (RSES) by gender and age-groups

RSES levels were significantly but marginally higher for men (*Mdn* = 36) than for women (*Mdn *= 34), *Z *=* *8.44, *P *<* *0.001, *r *=* *0.12. RSES total scores were also significantly different between the age groups, *H*(3) = 127.33, *P *<* *0.001, the older the higher values of Rosenberg scores (18–25: *Mdn *= 33; 26–39: *Mdn *= 34; 40–49: *Mdn *= 35; 50–65: *Mdn *= 36).

### Self-esteem and PT

The 90th percentiles of the LYLES-tot were set as a cut-off for PT, for women and men separately. RSES total scores were thereafter compared between PT and nPT, for women as well as for men. Comparisons were also made between women and men for PT and nPT.

In general there was a moderate effect of gender on self-esteem when comparing PT women and men, *Z *=* *6.33, *P *<* *0.001, *r *=* *0.27. PT women have lower RSES scores (Mdn = 30) than men (Mdn = 34). This effect was shown to be strong when analysing 18–25 year olds separately, *Z *=* *3.47, *P *<* *0.001, *r *=* *0.48. Note that there were only 11 men (Mdn = 35) compared to 42 women (Mdn = 25.5) in this comparison. Even though the small number of men might have introduced some random variation in the measures, the difference between women and men was nevertheless remarkable in terms of RSES scores. There was also a moderate effect of PT when comparing RSES scores for women only in this age group, *Z *=* *4.73, *P *<* *0.001, *r *=* *0.26, PT women have lower RSES scores (Mdn = 16) than nPT women (Mdn = 23). For all other comparisons the effect sizes were considered small and in some cases nonsignificant, Table 4.

**Table 4:** Comparisons of Rosenberg scores between poly (PT)- and nonpolytraumatized (nPT) (for men and women separately) and between men and women (for poly (PT)- and nonpolytraumatized (nPT) separately)

Age	Men (PT vs. nPT)	Women (PT vs. nPT)	PT (men vs. women)	nPT (men vs. women)
Tot				
*18*–*65*	*0.06*	*0.17*	*0.27*	*0.10*
18–25	0.10^ns^	0.26	0.48	0.09^ns^
26–39	0.02^ns^	0.15	0.25	0.08
40–49	0.14	0.18	0.21	0.11
50–65	0.08	0.16	0.21	0.07
nIPE				
*18*–*65*	*0.02*^ns^	*0.06*	*0.16*	*0.11*
18–25	0.02^ns^	0.06^ns^	0.13^ns^	0.15
26–39	0.03^ns^	0.02^ns^	0.24	0.09
40–49	0.08^ns^	0.06^ns^	0.11^ns^	0.12
50–65	0.05^ns^	0.07	0.12^ns^	0.08
IPE				
*18*–*65*	*0.09*	*0.17*	*0.21*	*0.10*
18–25	0.01^ns^	0.22	0.36	0.11
26–39	0.07^ns^	0.16	0.17	0.08
40–49	0.14	0.25	0.27	0.10
50–65	0.09	0.15	0.15	0.07
ACC				
*18*–*65*	*0.13*	*0.22*	*0.27*	*0.10*
18–25	0.06^ns^	0.18	0.27	0.11
26–39	0.13	0.20	0.20	0.08
40–49	0.16	0.25	0.20	0.10
50–65	0.12	0.21	0.26	0.07

nIPE, noninterpersonal event; IPE, Interpersonal event; ACC, adverse childhood circumstances; *r*, effect size; ns, nonsignificant (*P* > 0.05). Rosenberg scores for all ages (18-25) are in italics. Comparisons are reported by their corresponding effect size *r* (small: *r* ∽0.1; moderate *r* ∽0.3; large: *r* ∽0.5).

### RSES, SCL-25, and PT

**Figure 1:**
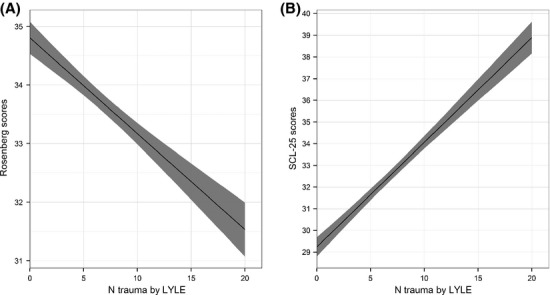
Linear relations between number (N) reported potential traumas and (A) scores on the Rosenberg self-esteem scale and (B) scores on SCL-25, including 95% confidence bands.

The number of reported potential traumas on LYLES-A showed almost linear relations with self-esteem (RSES decrease with increase of LYLES-A) and psychological distress (SCL-25 increase with increase of LYLES-A), see Fig.1.

## Discussion

This study has examined the prevalence of self-reported experiences of potential traumatic events (before the age of 18) using LYLES-A in a representative national sample of adults 18–65 years old and the association with psychological distress (SCL-25) and global self-esteem (RSES). Polytraumatization (PT) defined as the 90th percentile, or the 10% in the sample who reported the most frequent potential traumas, has been identified and also the association with psychological stress and self-esteem can be seen. The result can be summarized in five main findings.

*First,* having experienced at least one potentially traumatic event before the age 18 years is common, especially concerning noninterpersonal (n-IPE) traumatic life events with 92% reporting this experience - something that has been shown in several other studies (Arata et al. 2005; Richmond et al. 2009). Traumatic interpersonal (IPE) events were reported by approximately half of the population (44% women and 51% men), a figure rather close to what Richmond (Richmond et al. 2009) has reported. Finally, adverse childhood circumstances (ACC) were reported by around sixty percent (64% women and 59% men), which is more than reported by two earlier studies (Chiara and Straus 2008; Bellis et al. 2013) but about the same as what has been reported from the ACE-studies (Brown et al. 2009).

*Second,* cut-off values were identified for persons at the 90th percentile, the definition of PT, for the different LYLES-A scales. To be able to identify the 10% of the population who have experienced the most potential traumas is suggested by Finkelhor et al. (2009a) and has also been used in other studies (Soler et al. 2012, 2013). To identify this group can be considered important both for research and clinically, as this group has shown to be vulnerable to different physical and psychological difficulties (Anda et al. 1999, 2001, 2002; Dietz et al. 1999; Dube et al. 2001a,b, 2003; Dong et al. 2003, 2004) and could be a risk group for revictimization (Widom et al. 2008). However, consequences of PT experiences need to be further investigated.

*Third,* the impact of PT across the different aspects of potential traumas showed significant differences between men and women, with men reporting more experiences of potential traumas of all kinds - except for ACC, where no difference was found. Epidemiological studies in national samples concerning exposure to different sorts of traumas and gender differences are few, but Kessler et al. (1995) found that men were more exposed to trauma than women, 60% compared to 50%. They also highlighted that men and women are often exposed to different kinds of potential traumas but men are likely to experience almost every type of traumatic event - with the exception of sexual assault and rape. Regarding gender differences and ACC, no such differences have been reported from other studies; men and women report a similar prevalence of ACC as we have found here (U.S. Department of Health and Human Services 2010). This is something that can be understood in terms of there being no dissimilarities concerning gender and distribution of children in certain families, or exposure to divorce and also that boys and girls are equally exposed to bullying. Significant differences were found concerning educational level, with more reports of polytrauma from those having a lower educational level - something which has also been found in other studies (Chan et al. 2011). So education may be interpreted as being a protective factor*. Fourth,* the study also showed that women have higher SCL-25 scores than men, something which has also been found in other studies (Nettlebladt et al. 1993). People who have experienced PT have significantly higher values on SCL-25 than non-PT. However, these are significant values, with moderate to low effect sizes. Using the recommended cut-off for clinical psychiatric cases of ≥1.75 on SCL-25 (Nettlebladt et al. 1993; Strand et al. 2003) in the present study showed that there were significantly more persons with PT than with no PT, who scored above the cut-off with an effect size that could be considered as low to moderate.

*Fifth,* the impact of PT on global self-esteem measured by Rosenberg Self-Esteem Scale (RSES) was found significant, (*P* < 0.001) between men and women. Women with PT had lower RSES than men, with a moderate effect size (*r* = 0.27). For women in the age group 18–25 the global self-esteem was remarkably lower in the PT group, significant, (*P* < 0.001) and with strong effect size (*r* = 0.47). The strong effect size for women in this age group is a key finding. It must be seen as being of great importance to the helping professional and also of importance in understanding the difference between men and women. More women than men seek psychiatric help and as men often have been exposed to more traumas than women, it can be easy to overlook the greater impact PT has on women - who seem to be more vulnerable. Women's vulnerability in developing post traumatic symptoms compared to men, despite lower rates of trauma exposure, has been well-documented (Kessler et al. 1995; Tolin and Foa 2006). In a Spanish study it was found that global self-esteem could be seen as both a moderator and a mediating factor as a buffer against polyvictimization and mental health (Soler et al. 2013). However, the pathways to polytraumatization, for both men and women, need to be further investigated (Finkelhor et al. 2009b).

The almost linear association between self-reported PT and an increase in psychological distress, depression, and anxiety and at the same time a decrease in reported self-esteem is in line with previous research (Williams et al. 2007; Soler et al. 2012, 2013). This almost linear association between PT and an increase in SCL-25 scores must be taken seriously, as both anxiety and depression have detrimental effects on health and have also found to be associated with early death (Edmonson et al. 2013; Wedegaetner et al. 2013). Also self-esteem has, in studies, been shown to have an impact on mental health (Merianos et al. 2013) and has also, in other studies, shown to be associated with polyvictimization (Soler et al. 2012, 2013). These relationships need to be further examined.

It is worth taking into consideration the difference between men and women in respect of experienced PT; more women than men seek psychiatric help and their experienced potential traumas need to be taken seriously, and clinicians need to address this. The way to address polytrauma is still a matter of speculation, but cannot be neglected. It is essential that methods for routine screening and appropriate interventions are developed and implemented.

There is an aspect worth discussing, alongside studies in order to cover further aspects of this field, that Scott-Storey (2011) points out: that today there are many definitions that appear to describe almost the same concept, and she suggests that there is a need for research to clearly conceptualise and operationalize what is meant by polyvicitmization (Finkelhor et al. 2007a) lifetime polyvictimization (Finkelhor et al. 2009a) revictimization (Widom et al. 2008), polytraumatization (Gustafson et al. 2009), and cumulative trauma (Chiara and Straus 2008). It is also necessary to operationalize clearly what has been measured as potential trauma and adversity. We have, in this study, chosen to cover a broad spectrum of self-reported potential traumas – noninterpersonal and interpersonal, where also adverse childhood circumstances such as bullying and mental health in the family have been asked about. These aspects of potential traumatic experiences and difficult life events have, in separate studies, shown to be important for mental health. Noninterpersonal potential traumas can be seen as maybe not such a difficult experience, but natural disasters like the 2004 tsunami in Thailand can be a harrowing experience for many people (Wahlström et al. 2008). In a follow-up study in Sweden after the 2004 tsunami, in which many Swedes were struck by this disaster, it was shown that not only exposure to life threatening situations and losing people but also prior life events were related to an elevated risk of worsening mental health, as measured with the General Health Questionnaire (Wahlström et al. 2010).

A limitation in this study, even if the sample is large, is that the participation rate was only 53% of the population asked. Another limitation is the recall bias - especially when asking older people about what happened 40 years ago. Although what has been asked are often things people remember when questioned (Hardt and Rutter 2011). The cross-sectional character can be seen as a limitation. It is also possible that if we in this study had used questionnaires especially developed to identify symptoms related to experienced potential traumas, such as - for example -Trauma symptom Inventory-2 (Briere 2011) maybe the effect sizes, for instance, would have been stronger.

In this study, we have screened for multiple types of traumas in a national representative sample, something that there has been a lack of in previous research (Widom et al. 2008) and we have found no study looking at how experiences of polytrauma impact the sense of global self-esteem and psychological distress measured by SCL-25.

## Conflict of Interest

None declared.
