# Polytraumatization in an adult national sample and its association with psychological distress and self-esteem

**DOI:** 10.1002/brb3.344

**Published:** 2015-05-08

**Authors:** Doris Nilsson, Örjan Dahlstöm, Gisela Priebe, Carl Göran Svedin

In the Materials and Methods section, under the sub-headers of Questionnaires, Linköping's youth life events scale- adult, LYLES –A, the following was mentioned:

“The test–retest reliability has been found to be *r* = 0.79 (*P* < 0.01) and kappa statistics (Cohen's kappa) item per item range between 0.44 and 1.0 (Finkelhor et al. 2007b).”

The correct reference citation for this statement should be (Nilsson et al. 2010) and not (Finkelhor et al. 2007b).

We apologize for this error.

## References

[b1] Nilsson D, Dahlstöm Ö, Priebe G, Svedin CG (2015). Polytraumatization in an adult national sample and its association with psychological distress and self-esteem. Brain Behav.

